# Detection and Quantification of 5moU RNA Modification from Direct RNA Sequencing Data

**DOI:** 10.2174/0113892029288843240402042529

**Published:** 2024-04-16

**Authors:** Jiayi Li, Feiyang Sun, Kunyang He, Lin Zhang, Jia Meng, Daiyun Huang, Yuxin Zhang

**Affiliations:** 1 Wisdom Lake Academy of Pharmacy, Xi’an Jiaotong-Liverpool University, Suzhou, 215123, China;; 2 Department of Computer Science, Xi’an Jiaotong-Liverpool University, Suzhou, 215123, China;; 3 School of Information and Control Engineering, China University of Mining and Technology, Xuzhou, 221116, China;; 4 Department of Biological Science, Xi’an Jiaotong-Liverpool University, Suzhou, 215123, China

**Keywords:** Oxford nanopore sequencing, RNA modification, machine learning, 5-methoxyuridine, read-level modification profiling, *in vitro*-transcribed

## Abstract

**Background:**

Chemically modified therapeutic mRNAs have gained momentum recently. In addition to commonly used modifications (*e.g*., pseudouridine), 5moU is considered a promising substitution for uridine in therapeutic mRNAs. Accurate identification of 5-methoxyuridine (5moU) would be crucial for the study and quality control of relevant *in vitro*-transcribed (IVT) mRNAs. However, current methods exhibit deficiencies in providing quantitative methodologies for detecting such modification. Utilizing the capabilities of Oxford nanopore direct RNA sequencing, in this study, we present NanoML-5moU, a machine-learning framework designed specifically for the read-level detection and quantification of 5moU modification for IVT data.

**Materials and Methods:**

Nanopore direct RNA sequencing data from both 5moU-modified and unmodified control samples were collected. Subsequently, a comprehensive analysis and modeling of signal event characteristics (mean, median current intensities, standard deviations, and dwell times) were performed. Furthermore, classical machine learning algorithms, notably the Support Vector Machine (SVM), Random Forest (RF), and XGBoost were employed to discern 5moU modifications within NNUNN (where N represents A, C, U, or G) 5-mers.

**Results:**

Notably, the signal event attributes pertaining to each constituent base of the NNUNN 5-mers, in conjunction with the utilization of the XGBoost algorithm, exhibited remarkable performance levels (with a maximum AUROC of 0.9567 in the “AGTTC” reference 5-mer dataset and a minimum AUROC of 0.8113 in the “TGTGC” reference 5-mer dataset). This accomplishment markedly exceeded the efficacy of the prevailing background error comparison model (ELIGOs AUC 0.751 for site-level prediction). The model's performance was further validated through a series of curated datasets, which featured customized modification ratios designed to emulate broader data patterns, demonstrating its general applicability in quality control of IVT mRNA vaccines. The NanoML-5moU framework is publicly available on GitHub (https://github.com/JiayiLi21/NanoML-5moU).

**Conclusion:**

NanoML-5moU enables accurate read-level profiling of 5moU modification with nanopore direct RNA-sequencing, which is a powerful tool specialized in unveiling signal patterns in *in vitro*-transcribed (IVT) mRNAs.

## 1 INTRODUCTION

RNA modification is a crucial post-transcriptional process that plays a pivotal role in fine-tuning gene expression, mRNA stability, splicing, translation, and, ultimately, cellular function [1-5]. These chemical modifications encompass a wide array of covalent alterations, including but not limited to methylation, pseudouridylation, and acetylation, each adding an additional layer of complexity to the regulatory landscape [6-10]. Unravelling the intricacies of RNA modifications and deciphering their underlying regulatory mechanisms have emerged as fundamental endeavors within the domain of molecular biology [9, 11-15]. Consequently, these investigations hold the potential to provide valuable insights into disease pathogenesis and offer novel avenues for therapeutic intervention.

In particular, RNA modification is a crucial concern in the *in vitro* transcribed (IVT)-mRNA vaccine, which has emerged as a promising therapeutic modality for a variety of clinical applications due to its potential to deliver specific genetic information into target cells [16-19]. IVT-mRNA offers advantages, such as high specificity, low toxicity, and the ability to encode a wide range of therapeutic proteins [16, 20-22]. However, the immunogenicity of the IVT-mRNA vaccine can pose challenges to its clinical applicability, necessitating the development of strategies to enhance its stability and efficacy while minimizing adverse immune responses [23-25]. Uridine modifications, including Pseudouridine [Ψ] [26], N1-Methylpseudouridine [m1Ψ] [27], 5-methoxyuridine [5moU] [19], have been recognized as crucial elements in mRNA design to improve stability, translation efficiency, and immunogenicity profile. An aspect of considerable interest within the domain of enhancing the efficacy of the IVT-mRNA vaccine pertains to the strategic modification of uridine residues situated within the mRNA molecule [6, 28, 29]. Among the various uridine modifications mentioned, the 5-methoxyuridine (5moU) modification has newly garnered significant attention due to its potential to enhance the properties of IVT-mRNA [19]. The 5-methoxyuridine (5moU) modification is a chemical modification of RNA in which a methoxy group is added to the fifth carbon of the uridine base, which has also previously been reported in the database MODOMICS that contains information for 5moU RNA modification in native biological samples and its link to human disease [30]. In the context of IVT-mRNA, the 5moU modification has been associated with increased resistance to nuclease degradation, improved mRNA half-life, and reduced immunogenicity; therefore, accurate detection is crucial for the quality control of the IVT-mRNA vaccine [31, 32], thus boosting a better understanding of the impacts of the modification on mRNA stability, translation, and immune responses with the respective functional design of IVT-mRNA vaccine.

Nonetheless, the effective detection and quantification of 5moU modifications in IVT-mRNA remain challenging. Conventional next-generation sequencing (NGS) based experimental methods for 5moU detection include antibody-based methods [32, 33], chemical labelling methods [34], enzymatic conversion and detection [35], and mass spectrometry (MS) [36, 37]. The major limitation of the above methods in the case of therapeutic IVT mRNA studies is that they cannot provide read-level resolution. Fortunately, a third-generation platform, Oxford nanopore direct RNA sequencing, has emerged to address this issue [38-42]. This technique capitalizes on the detection of alternations in electric current as individual RNA molecules pass through nanopores, allowing for the reconstruction of nucleotide sequences based on these electrical signals and offering long reads that can cover the entire length of transcripts, thus enabling the preservation of epitranscriptomic information without the need for reverse transcription [43-47]. By analyzing the disruptions in electric signals when a modified base is present in the nanopore, these modifications can be identified by comparing the observed current with the reference [48-52].

**Fig. (1):**
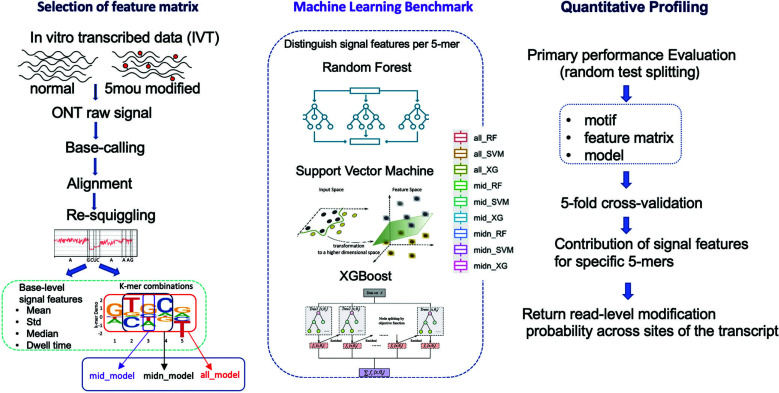
The schematics of the proposed framework. The framework consists of a pre-processing procedure (left), implementation of machine learning models, and respective feature matrices (middle). Then, the 5-mer-specific performances were further evaluated (right).

In 2021, ELIGOS presented the first, which is also the only published ONT-based 5moU prediction tool until now. This method relies on statistical comparison of site-level base call error profiles in target and unmodified control datasets [53]. Landscapes of modifications on individual molecules cannot be known in this case. Also, a control sample is required. Here, we presented a novel machine-learning-driven 5moU detection tool that enables de novo (*i.e*., no control sample required) and read-level analysis. We utilized signal features (*i.e*., current intensities mean, standard deviation, median, and dwell time) extracted from 100% modified and unmodified *in vitro*-transcribed (IVT) data. Classical machine learning algorithms, including Random Forest (RF), Support Vector Machine (SVM), and XGBoost, were used to train 5-mer specific models (Fig. **1**). 5-mer signal features, plus the XGBoost algorithm, achieved exceptional performance for read-level modification detection (maximum AUROC = 0.9567 for the AGTTC reference 5-mer dataset and minimum AUROC = 0.8113 for the TGTGC reference 5-mer dataset), which surpassed the existing background error comparison models (ELIGOs; AUC= 0.751 for 5moU IVT dataset) [53].

## 2 MATERIALS AND METHODS

### 2.1 Data Collection

Direct RNA sequencing samples of both 100% 5moU modified and unmodified samples from ELIGOS [53] were collected. These two samples were *in vitro* transcribed using the luciferase gene as a template. Detailed information on the template can be found in the Supplementary Information. Due to the lack of publicly available, reliable data sources, we failed to showcase the feasibility of our workflow with external validation. As an alternative, we developed a unique dataset allowing for modification rate customization to mirror diverse data distributions more closely. This includes creating a custom validation dataset from the original 100% 5moU modified and 0% modified samples, with mixed data ratios of 8:2, 2:8, 6:4, and 4:6 (modified: unmodified).

### 2.2 Basecalling, Mapping, and Re-squiggle

The raw sequencing data, initially provided in the FAST5 format, underwent base-calling using the Guppy software (version 2.3.4) [54, 55]. In order to uphold the quality of the dataset, only reads surpassing a minimum length of 200 bases were subjected to subsequent analysis. Qualified reads were aligned to reference sequences using Minimap2 (version 2.17) [56], and the resulting alignment file was sorted, compressed, and indexed with Samtools (version 1.17) [57]. Then, the re-squiggle module of the Tombo software [58] (version 1.5.1) was used to define an assignment from read signal to reference based on the alignment [59] using the signal assignment algorithm. Re-squiggle information was included in the FAST5 files downloaded from ELIGOS data.

### 2.3 Signal Feature Extraction

The extraction of signal features from FAST5 files was undertaken by employing a customized Python script modified from previous work on RNA modification detection from direct RNA sequencing data using machine learning and deep learning [60, 61]. The pursuit involved the identification of the NNUNN 5-mer (where N represents A, C, U, or G. Note that there are uses of “T” instead of “U” in subsequent content since the base-caller outputs sequences with “T”, which represents “U” in RNA samples), from which we proceeded to extract pivotal statistical parameters. For each NNUNN 5-mer along the read, current event mean (denoted as mean_1~mean_5), median (denoted as mdintense_1 ~mdintense_5), standard deviation (denoted as sd_1~sd_5), and dwell time (denoted as L-1~L-5) for each base of the 5-mer were calculated and extracted (Supplementary Fig. **S1** for a demonstration of the generated feature matrix). Prior to model implementation, the Mann-Whitney test [62-64], a non-parametric statistical method, was utilized to quantify the level of difference between modified and normal 5-mer samples for all 20 features respective to 5 positions.

### 2.4 Machine Learning Approach

In this machine learning tool development, the goal is to classify 5moU in a certain dataset and return the probability of the read being modified for an unknown sample with a mixture of 5moU modified and normal samples, from which the ultimate and general goal is to reach modification detection using Oxford nanopore RNA sequencing pipeline for IVT-mRNA quality control. With respect to every expressed 5-mer sequence, ensemble attributes sourced from both modified (positive) and unmodified (negative) samples were meticulously categorized, amalgamated, and subsequently partitioned into distinct training and testing subsets. We then employed three decent binary classification algorithms: Support Vector Machines (SVM) [65], Random Forest (RF) [66], and XGBoost [67-70]. Models were implemented by the Python scikit-learn package (version 1.7.6). The model parameters were optimized through 5-fold cross-validation during the learning stage.

### 2.5 Evaluation Metrics

The classification performance was measured using the receiver-operating characteristic (ROC) and evaluated based on the area under the ROC curves (denoted as AUROC or AUC). Additionally, we employed commonly adopted assessment metrics, such as accuracy (Acc), precision (Pr), recall (Re), F1 score (F1), and Matthew’s correlation coefficient (Mcc), to further evaluate the model performance (Eqs. 1-4) [71-73].


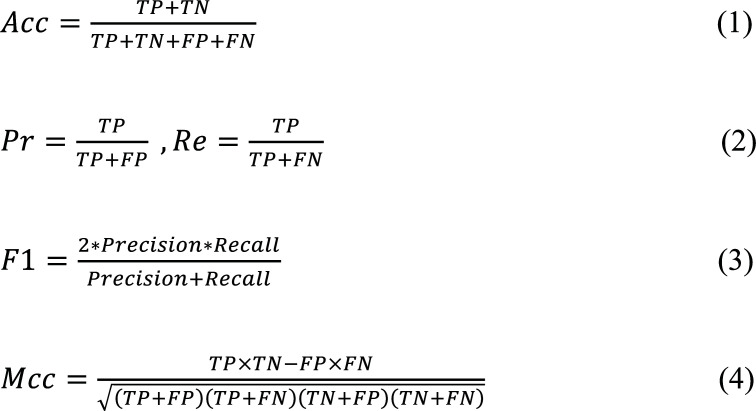


Where TP, TN, FP, and FN represent the number of true positive, true negative, false positive, and false negative, respectively.

## 3 RESULTS AND DISCUSSION

### 3.1 5moU RNA Modification Causes Significant Alternation in ONT Signals

**Fig. (2):**
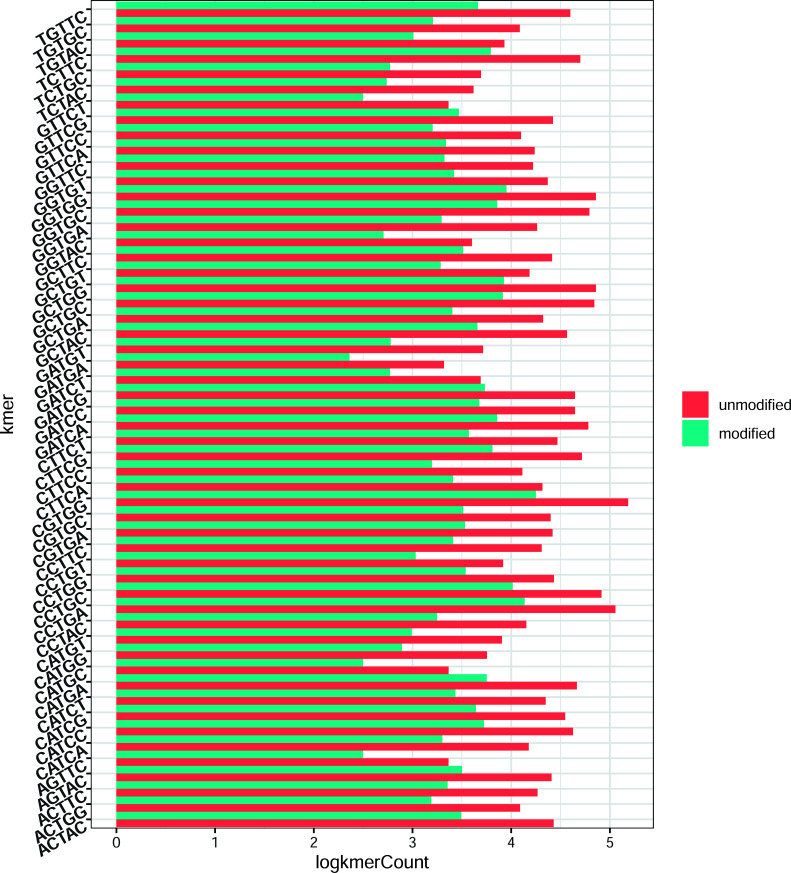
K-mer occurrences (logkmerCount) in unmodified and modified samples across the given transcript. A total of 1822824 5-mer samples that contain a U / 5mou base in the middle are obtained: 197147 modified and 1625677 unmodified samples, with a total of 54 different model 5-mer combinations out of the possible 256 combinations. Note that the base “T” in the k-mer represents the base “U” in the RNA sequence samples.

A total of 54 out of the possible 256 5-mers were expressed from the luciferase gene template (Fig. **2**). Differences in counts of diverse 5-mer motifs and the number of modified/unmodified samples could be considered implicit features in the base-calling, alignment, and mapping process, partially representative of the information of background error, which is a more encapsulated version of the benchmark dataset in Eligos2 RNA background error model (rBEM) [53]. Subsequently, each model 5-mer was trained and tested in parallel to derive motif-specific performance under various combinations of feature matrix and machine learning model.

**Fig. (3):**
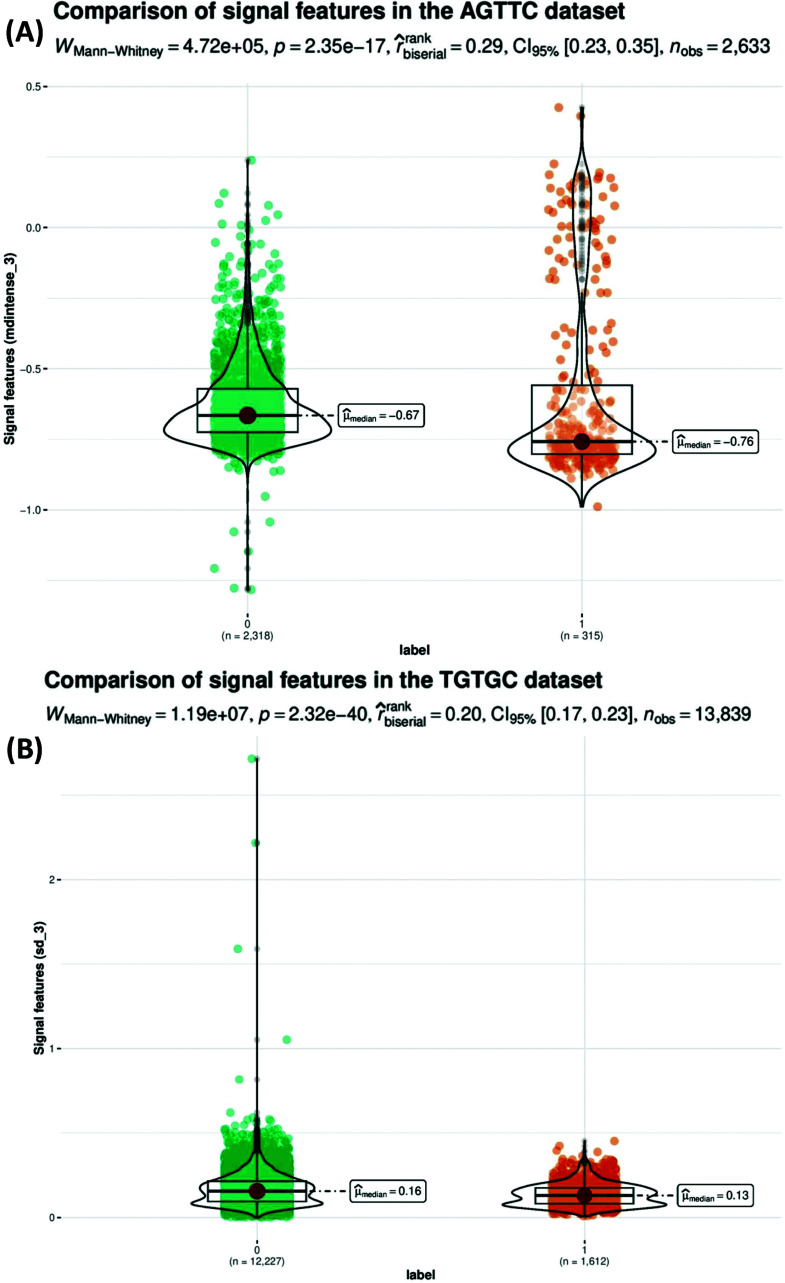
Selected visualizations of the Mann-Whitney non-parametric test results for the centre U position. Comparison of signal features to show the signal alternation caused by modification, from which the feature with the most significant difference in the centre U position between unmodified (labelled as 0) and modified (labelled as 1) is shown. (**A**) Results for the AGTTC dataset. (**B**) Results for the TGTGC dataset.

In order to elucidate the extent of dissimilarity between unmodified and 5moU-modified samples, specifically concerning the compilation of 20 signal features spanning 5 positions within each 5-mer sequence, a rigorous assessment was undertaken. This evaluation encompassed the application of the Mann-Whitney non-parametric test [62] to the AGTTC and TGTGC subsets, the former yielding the highest efficacy and the latter demonstrating the least performance within the testing phase (Fig. **3** and Supplementary Fig. **S2**). The test results indicated a statistically significant difference between the two groups (*p* < 0.05). This suggests that there is evidence to support the hypothesis that the signal distributions of the normal and 5moU modified samples differed significantly in both selected datasets. The effect size was also calculated using the Mann-Whitney test, typically reported as W, which provides an estimate of the magnitude of the difference between the groups. A larger W value indicates a larger effect size, indicating that the two groups are distinct in terms of the variables being tested.

### 3.2 Enhancing Model Capabilities through Signal Features from Adjacent Positions

**Fig. (4):**
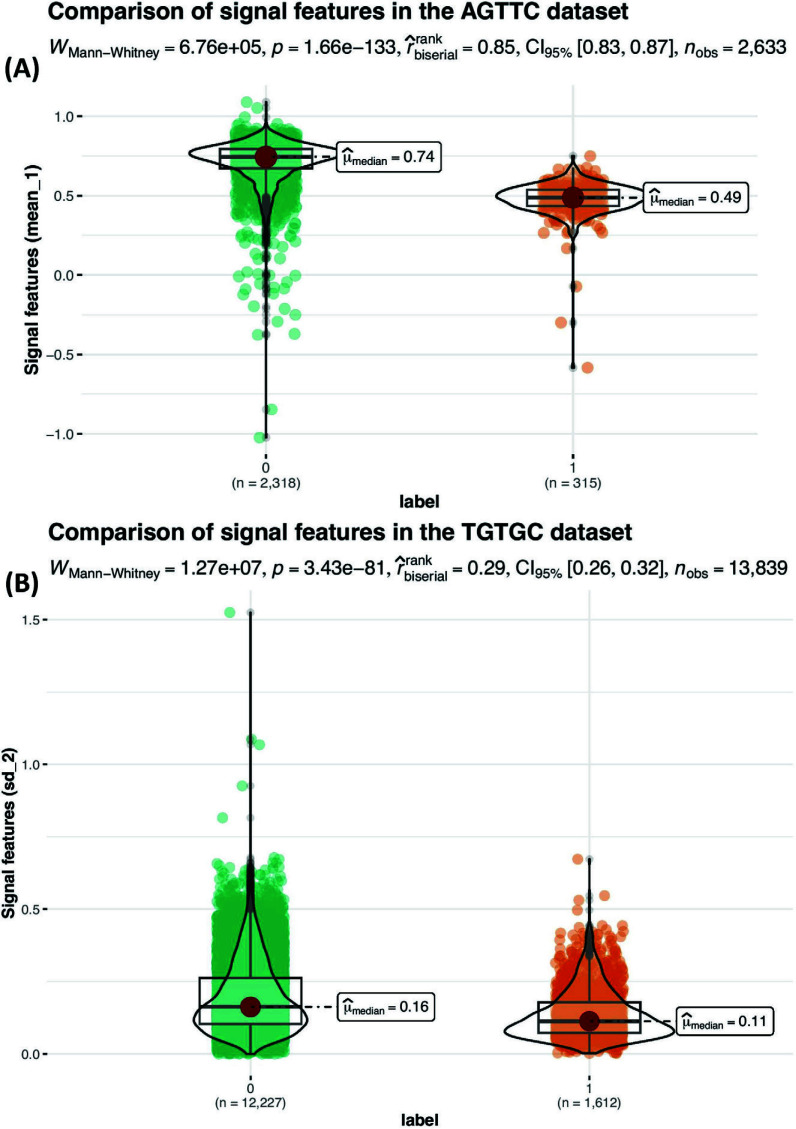
Selected visualizations of the Mann-Whitney non-parametric test results for neighbouring positions. Comparison of signal features to show the signal alternation caused by modification, from which the feature with the most significant difference in neighbouring bases between unmodified (labelled as 0) and modified (labelled as 1) is shown. (**A**). Results for the AGTTC dataset. (**B**). Results for the TGTGC dataset.

Under the general workflow, the feature extraction and primary performance comparison process were classified into two broad categories. The Supplementary Fig. **S1** contains a demonstration of the generated feature matrix with the mentioned column names of corresponding signal features. The first one considers the 5mer as a unit passing through the pore and taking all the twenty feature columns of four signal numerical features respective to five positions in specific 5-mers in the dataset (denoted as “all_RF”, “all_SVM”, “all_XG”); the second one considers the middle position as the target for 5moU modification; thus, it only takes signal features of the middle position in each 5-mer (denoted as “mid_RF”, “mid_SVM”, “mid_XG”); the third one considers the middle and one neighbouring position surrounding it, resulting in 15 feature columns (denoted as “midn_RF”, “midn_SVM”, “midn_XG”). The subdivision of the “all_model” category was compared using specific 5-mers that were sampled from 5-mers respective to different AUC values. It is worth noticing that signal features of neighbouring positions in 5-mers are also informative besides those of the middle position. Signal alternations on the neighbouring bases were justified by the results of the Mann-Whitney non-parametric test, demonstrating the signal features with the most significant alternations in neighbouring positions (Fig. **4**). Fig. (**5**) presents the “all_model” feature matrix for the three baseline models, which shows significantly higher AUC values than the “mid_model” and “midn_model” feature selection schemes. The XGBoost model generally shows the highest AUC value compared to the other two models, from which the 5-mer with the highest AUC is AGTTC and the lowest is TGTGC, which is consistent with the three machine learning models.

### 3.3 Machine Learning Algorithm Benchmark

An exhaustive assessment was conducted to ascertain the viability of widely employed binary classification machine learning algorithms. The outcomes of a comprehensive 5-fold cross-validation procedure for models that exhibited the most exceptional and the least satisfactory performance are tabulated in Tables **1** and **2**. Additionally, the Receiver Operating Characteristic (ROC) and Precision-Recall (PR) curves related to these analyses are visually represented in Fig. (**6**). According to the results, we can derive that the XGBoost indeed manifests exceptional performance after rational parameter tuning, with an average AUC of 0.9567 in the AGTTC dataset and 0.8113 in the TGTGC dataset. Additionally, the feature contribution of the XGBoost model visualized by the SHAP module [74, 75] could be analyzed from Supplementary Fig. (**S3**), with insightful information considering differences in feature contribution of signal features across positions of the 5-mer to the output of the classifier.

For figure legends in plots (**A-D**), “SVC” represents Support Vector Machine (SVM), “RandomForestClassifier” is abbreviated as “RF”, “XGBClassifier” represents XGBoost (abbreviated as XGB or XG).

In an effort to unveil the site-level modification status of a given IVT-mRNA transcript and further demonstrate the ability of the three classifiers in classifying 5moU and normal uridines, predicted probabilities derived from three classifiers on the independent validation data were subjected to a comprehensive comparison supplemented by a visual representation in Fig. (**7**). In the data curation section, we separated an additional validation set to be excluded from the training and test stages in the further stage of model development, from which the modification status of those separated samples was determined. The AGTTC and TGTGC datasets, which consisted of additional validation samples with known modified and unmodified attributes, were utilized as the reference for this evaluation. In alignment with the findings reported in the preceding sections, the AGTTC dataset generally exhibited better performance than the TGTGC dataset, from which the XGBoost model demonstrated superior performance among the three classifiers in both datasets. The XGBoost model returned high probability values for 5moU modified reads and low probability values for normal reads in a more accurate pattern than the other two models. These validation results, in conjunction with a comprehensive evaluation across different feature selection matrices and model settings, underscore the NanoML-5moU framework's potential as a robust tool for detecting 5moU modifications in IVT samples through the Oxford Nanopore direct RNA sequencing workflow.

### 3.4 Model Applicability in Datasets with Varied Modification Ratios

Within the overarching methodology of our framework, the ability to infer the modification status of individual reads from extracted signal features has been established. This approach has demonstrated that the proposed machine learning benchmarks are both practical and effective in distinguishing between modified and unmodified samples, with a particular focus on IVT data patterns. To further explore the potential widespread applicability of the NanoML-5moU workflow, it is envisaged that additional sequencing samples containing 5moU RNA modifications will be incorporated for external validation. Up to the present, direct RNA sequencing samples, including both 100% 5moU modified and unmodified samples from ELIGOS, have been collected to serve as the dataset for our proposed workflow. Currently, there is an absence of such data sources that are systematically reliable and publicly accessible. However, this study demonstrated the framework's capability to be applied across diverse data patterns. Specifically, we have compiled a novel dataset collection that allows for the customization of modification rates to reflect diverse data distributions more accurately. Originating from samples with 100% 5moU modification and 0% modification, a new customized validation dataset was developed, featuring mixed data of the transcript with modification to unmodified ratios of 8:2, 2:8, 6:4, and 4:6, respectively.

The efficacy of the NanoML-5moU framework was further validated through additional experiments using the newly curated collection of mixed data with varying modification ratios. A comparative analysis of the performance of the proposed workflow is presented in Fig. (**8**). Within each subset of the new dataset collection, the optimal set of feature matrix selections, namely, ‘all_XG’, ‘all_RF’, and ‘all_SVM’, were implemented and compared. Consistent with the findings from the Machine Learning algorithm benchmark section, all three machine learning methods exhibited significant performance, with AUROC values exceeding 0.8, where the XGBoost method consistently achieved superior performance. These findings, coupled with the performance comparison under various feature selection matrices and model settings, highlight the NanoML-5moU framework as a potentially powerful and specialized benchmark for the detection of 5moU modifications in IVT samples through the Oxford Nanopore direct RNA sequencing workflow. This framework is proven to be accurate and robust, capable of specifically distinguishing read-level signal features.

## CONCLUSION

Chemically modified synthetic mRNA therapeutics have gained momentum in recent years. In addition to well-studied m6A and pseudouridine, 5moU is recognized as a promising substitution for normal nucleotide. In this case, identification and quantification of 5moU would be critical for future therapeutic mRNA design and quality control. By harnessing the inherent capabilities of nanopore direct RNA sequencing, the profiling of modifications from native mRNAs can be seamlessly achieved, obviating the necessity for reverse transcription procedures. ELIGOS, as the only ONT-based 5moU detection tool, cannot provide modification status on individual reads. Within the scope of this study, we have endeavoured to bridge the existing lacuna pertaining to the read-level prediction of 5-methoxyuridine (5moU) modification, which has been facilitated through the strategic utilization of nanopore single-molecule direct RNA sequencing techniques.

To fully capture and exploit the intrinsic and insightful signal features of the 5moU modified samples, the Mann-Whitney non-parametric statistical test was implemented to quantify the degree of difference between signal features of 5moU modified and normal unmodified samples. The outcomes elucidated that the incorporation of 5moU induces discernible alterations in the signal patterns not only at the modified position but also in its proximal neighbouring positions. The framework integrated various baseline machine learning algorithms, including SVM, RF, and XGBoost, and utilized signals extracted from nanopore signals. Results demonstrated the exceptional performance of the XGBoost method, with the maximum AUROC = 0.9567 achieved in the AGTTC dataset and the minimum AUROC = 0.8113 in the TGTGC dataset. This outcome notably surpassed the performance of the established background error comparison model, indicated by an Area Under the Curve (AUC) value of 0.751 in ELIGOs for site-level prediction.

Although nanoML-5moU is capable of detecting and quantifying 5moU modification at read-level with exceptional performance, the validity of the proposed framework still relies on the improvement of pre-processing software tools that map the raw sequencing signals to the benchmark datasets. With the advancement of nanopore sequencing protocols and base-calling algorithms, significant enhancements in read mapping accuracy and nanopore signal association are anticipated. Concomitant enhancements, in conjunction with the simultaneous advancement of deep learning architectures for nanopore base-calling, hold the promise of refining existing base-calling models. As a corollary, such refinement would inevitably engender the creation of RNA modification detection tools characterized by heightened precision and accuracy.

It is our perspective that the NanoML-5moU framework assumes a pivotal role in accurately identifying 5moU modification from direct RNA sequencing of therapeutic IVT-mRNA, particularly in the context of mRNA vaccine quality control procedures. Moreover, the framework possesses the potential for extension to encompass the identification of various additional RNA modifications within both IVT RNA and native biological samples, which hold a pivotal significance in the realm of mRNA vaccines and exhibit intrinsic associations with human diseases.

## Figures and Tables

**Fig. (5):**
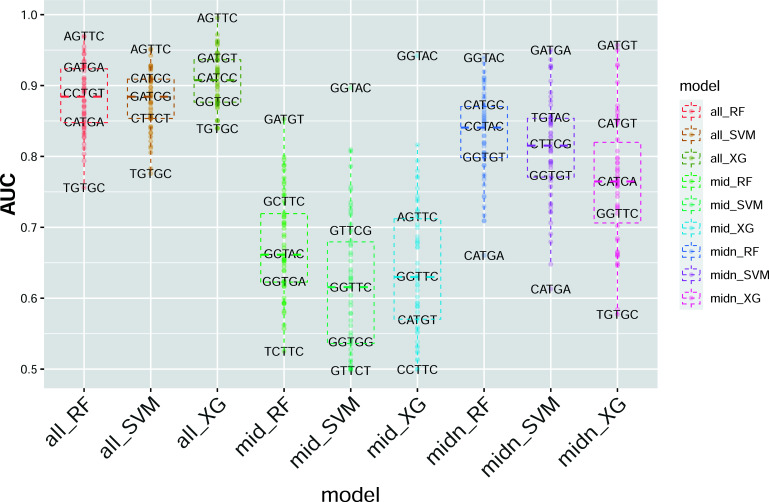
AUC comparison for different 5-mer datasets within feature-model groups. The 9 groups in the x-axis represent the combinations of the feature matrix and model implemented, from which the 54 diverse 5-mer datasets are trained and tested in parallel. Each data point within the specific group denotes the AUC value of the associated 5-mer dataset.

**Fig. (6):**
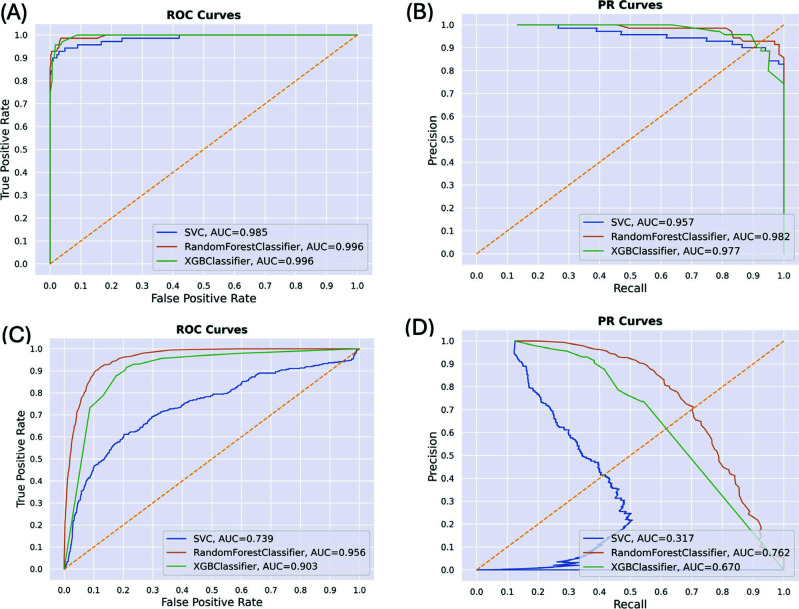
ROC and PRC for selected 5-mers with the best (“AGTTC”) and the worst (“TGTGC”) performance. (**A**) ROC curves for the AGTTC dataset. (**B**) PR curves for the AGTTC dataset. (**C**) ROC curves for the TGTGC dataset. (**D**) PR curves for the TGTGC dataset.

**Fig. (7):**
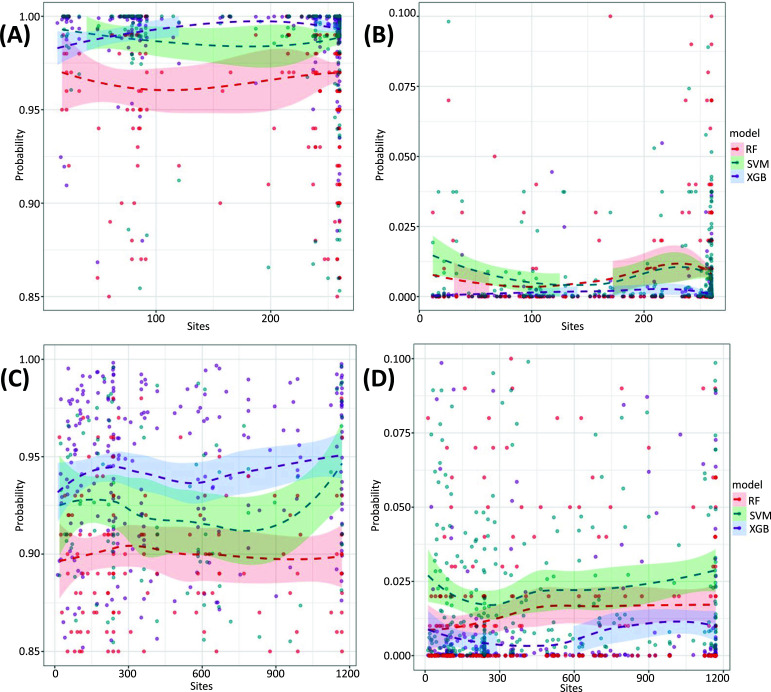
Visualization of the read-level modification probability across sites of the given transcript based on three machine learning models. (**A-B**). Results for the AGTTC dataset, with modified (**A**) and unmodified (**B**) samples as reference, respectively. (**C**-**D**). Results for the TGTGC dataset, with modified (**C**) and unmodified (**D**) samples as reference, respectively. Note that for the comparison between different models, cut-offs on the y-axis were chosen, which are the interval (0.85, 1.00) and the interval (0,000, 0.100) for modified and unmodified reference samples, respectively, where most of the points aligned in the mentioned interval. A complete set of plots with full y-axises are available in Supplementary Fig. (**S4**) for the AGTTC dataset and Supplementary Fig. (**S5**) for the TGTGC dataset.

**Fig. (8):**
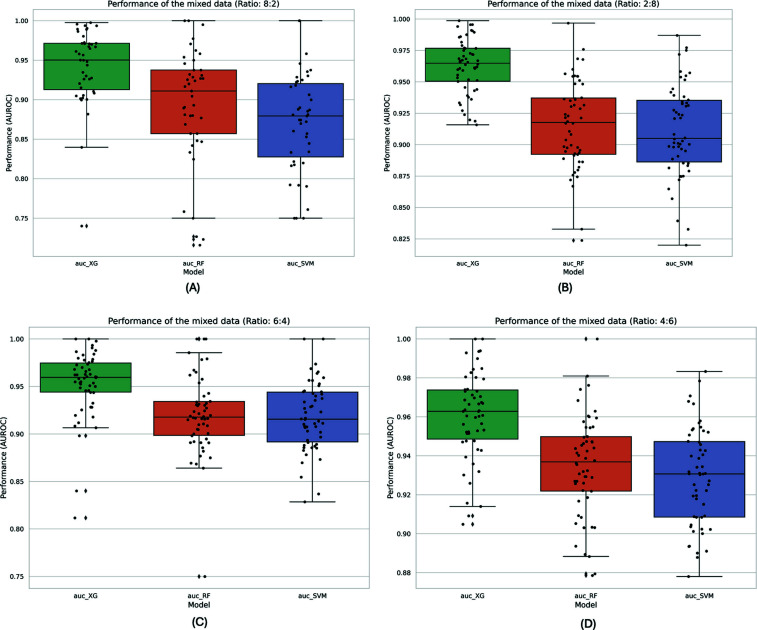
Boxplot visualization of the model performance on newly curated datasets with customized mixed ratios. AUROC performance of XGBoost (XG), Random Forest (RF), and Support Vector Machine (SVM) is shown in each of the four figures under different mixed ratios in the curated dataset. Originating from samples with 100% 5moU modification and 0% modification, a novel, customized validation dataset was developed, incorporating mixed data with modification to unmodified ratios of 8:2, 2:8, 6:4, and 4:6, respectively. (**A-B**). Model performance on curated datasets with ratios of 8:2 and 2:8, respectively. (**C-D**). Model performance on curated datasets with ratios of 6:4 and 4:6, respectively.

**Table 1:** 5-fold cross-validation results for the “AGTTC” dataset.

**Model**	**AUROC**	**AUPRC**	**ACC**	**Precision**	**Recall**	**F1 Score**	**Mcc**
**XGBoost**	**0.9567** **+/-** **0.007**	0.9810 +/- 0.0074	0.9852 +/- 0.0025	0.9544 +/- 0.0339	0.9567 +/- 0.0070	0.9358 +/- 0.0143	0.9280 +/- 0.0158
Random forest	0.9489 +/- 0.0087	0.9818 +/- 0.0064	0.9852 +/- 0.0022	0.9715 +/- 0.0108	0.9489 +/- 0.0087	0.9349 +/- 0.0115	0.9275 +/- 0.0121
SVM	0.9003 +/- 0.0067	0.9328 +/- 0.0205	0.9704 +/- 0.0037	0.9344 +/- 0.0508	0.9003 +/- 0.0067	0.8656 +/- 0.0199	0.8525 +/- 0.0232

**Table 2:** 5-fold cross-validation results for the “TGTGC” dataset.

**Model**	**AUROC**	**AUPRC**	**ACC**	**Precision**	**Recall**	**F1 Score**	**Mcc**
**XGBoost**	**0.8113** **+/-** **0.0156**	0.7746 +/- 0.0252	0.9338 +/- 0.0053	0.7472 +/- 0.0314	0.8113 +/- 0.0156	0.6960 +/- 0.0278	0.6611 +/- 0.0305
Random forest	0.7689 +/- 0.0101	0.7478 +/- 0.0242	0.9298 +/- 0.0030	0.7756 +/- 0.0249	0.7689 +/- 0.0101	0.6494 +/- 0.0156	0.6219 +/- 0.0159
SVM	0.6196 +/- 0.0139	0.3400 +/- 0.0568	0.9033 +/- 0.0154	0.7667 +/- 0.2906	0.5196 +/- 0.0139	0.0763 +/- 0.0485	0.1576 +/- 0.0848

## Data Availability

The data and supportive information are available within the article (Code availability: https://github.com/JiayiLi21/NanoML-5moU).

## LIST OF ABBREVIATIONS

5moU: 5-methoxyuridine
AUC: Area Under the Curve
IVT: *In Vitro*-transcribed
MS: Mass Spectrometry
NGS: Next-generation Sequencing
PR: Precision-recall
RF: Random Forest
ROC: Receiver-operating Characteristic
SVM: Support Vector Machine
