# Supplementary material for: Detection and Quantification of 5moU RNA Modification from Direct RNA Sequencing Data
Source: Curr Genomics. 2024 Apr 16;25(3):212–25. doi: 10.2174/0113892029288843240402042529 (PMC11288159; doi:10.2174/0113892029288843240402042529)

Supplementary Material

Detection and Quantification of 5moU RNA Modification from Direct RNA Sequencing Data

Jiayi Li[1], Feiyang Sun[2], Kunyang He[1], Lin Zhang[3], Jia Meng[4], Daiyun Huang[1] and Yuxin Zhang[4,*]

[1]Wisdom Lake Academy of Pharmacy, Xi'an Jiaotong-Liverpool University, Suzhou, 215123, China; [2]Department of Computer Science, Xi'an Jiaotong-Liverpool University, Suzhou, 215123, China; [3]School of Information and Control Engineering, China University of Mining and Technology, Xuzhou, 221116, China; [4]Department of Biological Science, Xi'an Jiaotong-Liverpool University, Suzhou, 215123, China

Supplementary Information for Data Collection.

Data Sources: https://trace.ncbi.nlm.nih.gov/Traces/index.html?view=study&acc=SRP166020

Raw data used in this project:

5moU 100% modified samples-fast5 file :https://sra-pub-src-1.s3.amazonaws.com/SRZ190756/LUC_5mou_dRNA_fast5.tar.gz.1

normal unmodified samples-fast5 file: https://sra-pub-src-1.s3.amazonaws.com/SRZ190757/LUC_normal_dRNA_fast5.tar.gz.1

Figure S1. A demonstration of the generated feature matrix

Under the general workflow, the feature extraction and primary performance comparison process is developed into two broad categories, the first one considers the 5mer as a unit passing through the pore and taking all the twenty feature columns of four signal numerical features respective to five positions in specific 5-mers in the dataset (denoted as “all_RF”, “all_SVM”, “all_XG”); the second one considers the middle position as the target for 5mou modification thus only takes signal features of the middle position in each 5-mer (denoted as “mid_RF”, “mid_SVM”, “mid_XG”); the third one considers the middle and one neighbouring position surrounding it, resulted in 15 feature columns（denoted as “midn_RF”, “midn_SVM”, “midn_XG”. The subdivision of the “all_model” category will be compared in the next section using specific 5-mers that are sampled from 5-mers respective to different AUC values.

	indx	kmer	mean_1	mean_2	mean_3	mean_4	mean_5	std_1	std_2	std_3	...	mdintense_2	mdintense_3	mdintense_4	mdintense_5	L-1	L-2	L-3	L-4	L-5	label
**0**	20	CATCT	-0.261382	-0.290529	-0.375176	-0.548695	-0.420694	0.034490	0.048193	0.075762	...	-0.302072	-0.405521	-0.548282	-0.413797	6	19	12	50	6	1
**1**	22	TCTTC	-0.375176	-0.548695	-0.420694	-0.102165	0.324141	0.075762	0.072908	0.065934	...	-0.548282	-0.413797	-0.103449	0.339314	12	50	6	29	12	1
**2**	23	CTTCG	-0.548695	-0.420694	-0.102165	0.324141	0.612248	0.072908	0.065934	0.077537	...	-0.413797	-0.103449	0.339314	0.643455	50	6	29	12	24	1
**3**	35	CGTGG	-0.134270	0.017096	0.317245	0.286391	0.191036	0.240493	0.116132	0.178935	...	0.000000	0.318624	0.264830	0.208968	29	38	6	19	6	1
**4**	44	CCTGC	-1.068734	-0.164691	0.164829	0.237456	-0.655237	0.200275	0.057011	0.052036	...	-0.165519	0.161381	0.240002	-0.657938	131	35	18	39	72	1
**...**	...	...	...	...	...	...	...	...	...	...	...	...	...	...	...	...	...	...	...	...	...
**1822819**	350	GCTGG	0.160716	0.201952	0.291149	0.341133	0.261907	0.102909	0.062878	0.116291	...	0.194064	0.291096	0.343161	0.269796	22	6	980	14	6	0
**1822820**	365	GATCC	0.713934	0.005522	-0.667390	-0.689873	-0.575091	0.217518	0.459278	0.056848	...	0.030766	-0.688690	-0.655557	-0.570358	6	6	6	16	6	0
**1822821**	374	GATCC	0.404221	0.204545	-0.725696	-0.713145	0.062983	0.418295	0.532178	0.098837	...	0.508826	-0.731289	-0.707623	0.059166	10	7	2203	6	625	0
**1822822**	377	CCTGA	-0.713145	0.062983	0.264506	0.270585	0.073087	0.044437	0.174422	0.070813	...	0.059166	0.243763	0.250863	0.134898	6	625	34	6	17	0
**1822823**	380	GATCA	0.270585	0.073087	-0.577806	-0.538803	0.255019	0.056049	0.242040	0.213394	...	0.134898	-0.627157	-0.579825	0.257963	6	17	211	6	82	0

1822824 rows × 23 columns

Detailed information regarding the code and data for this framework are available on the GitHub reposito-ry(https://github.com/JiayiLi21/NanoML-5moU/tree/main)

Figure S2 Mann-Whitney non-parametric test results for AGTTC and TGTGC in terms of 20 signal features

1. AGTTC results

1. (a) AGTTC dataset: Signal features in the first position

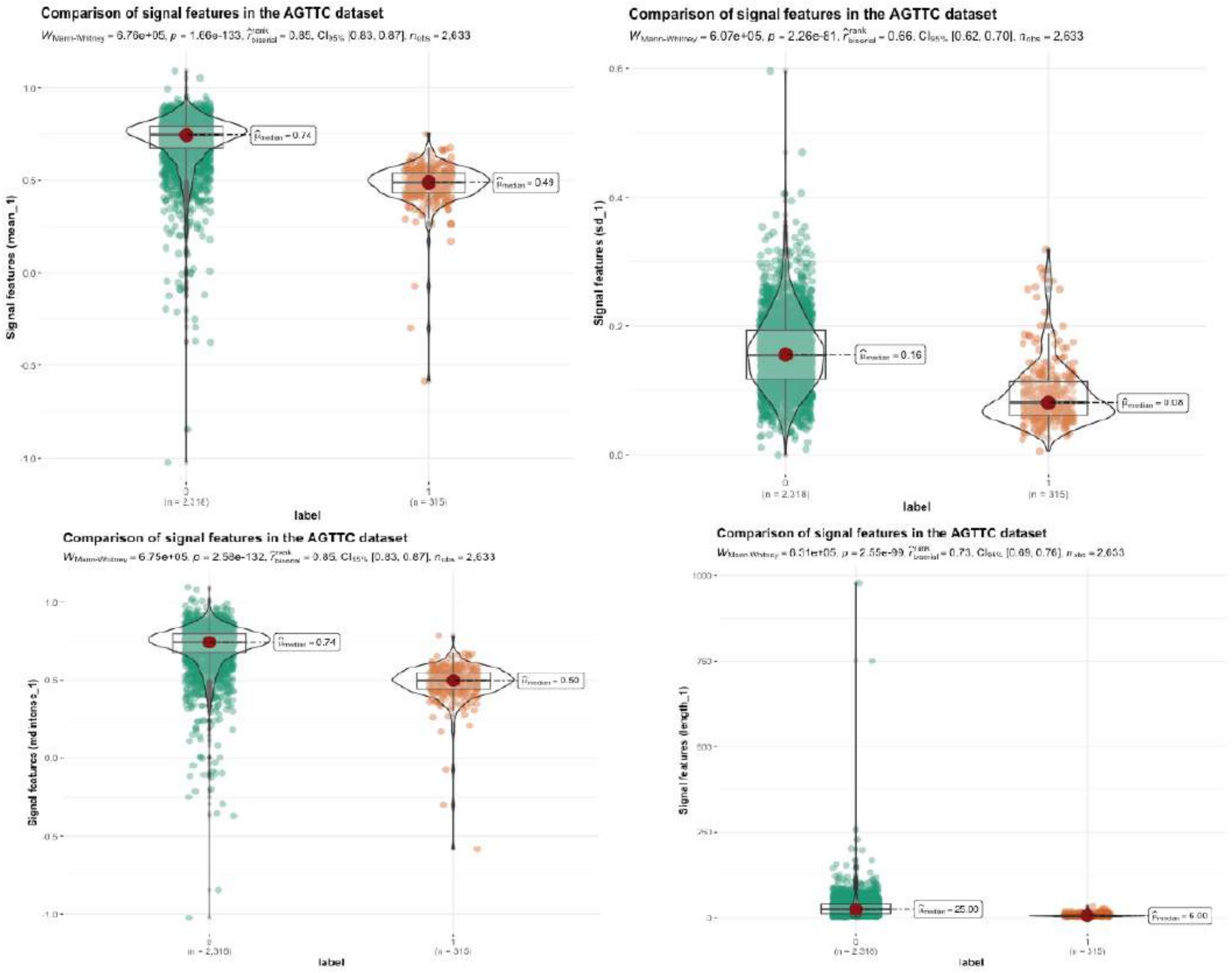

Figure S2

1. (b) AGTTC dataset: Signal features in the second position

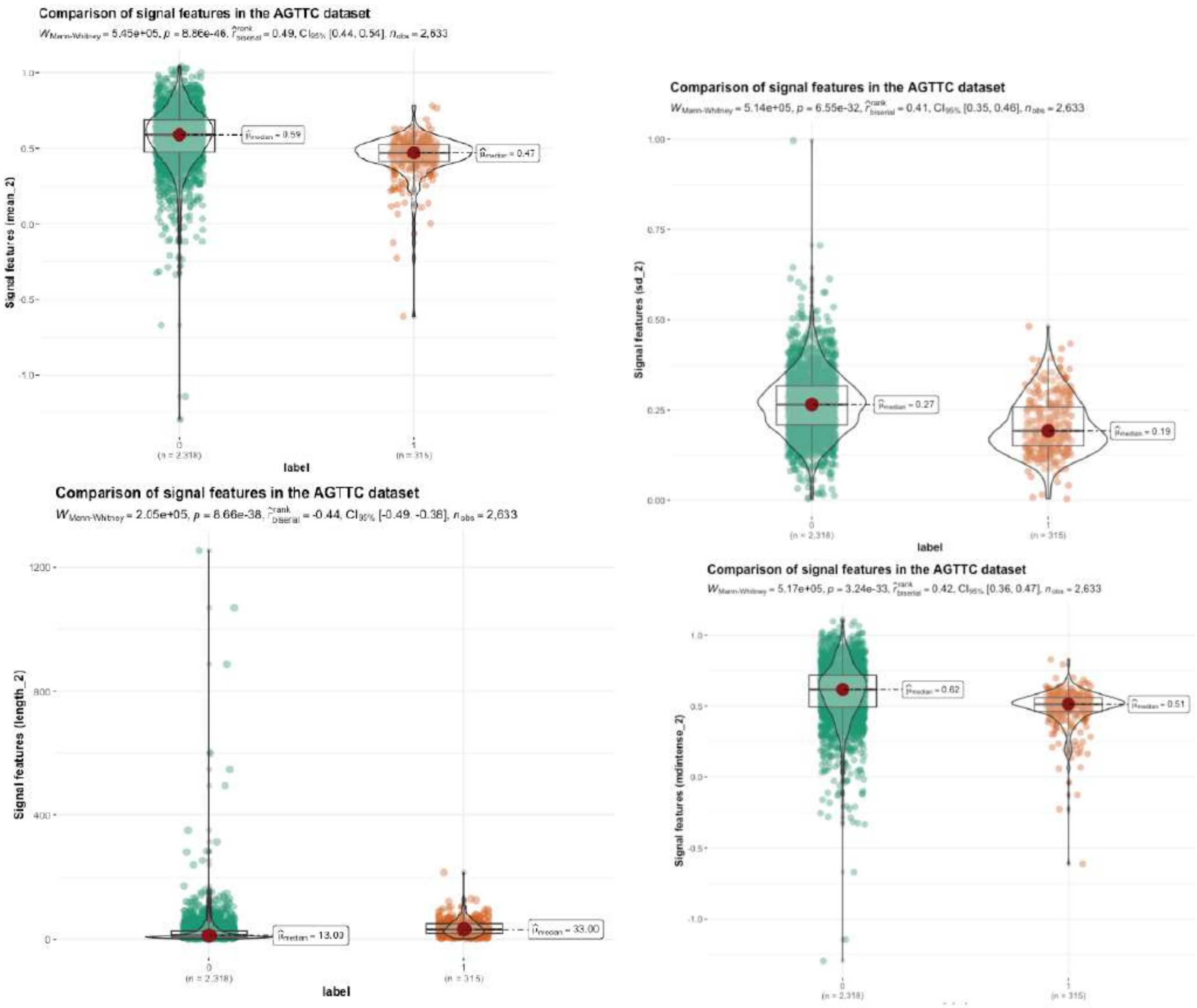

Figure S2

1. (c) AGTTC dataset: Signal features in the third position

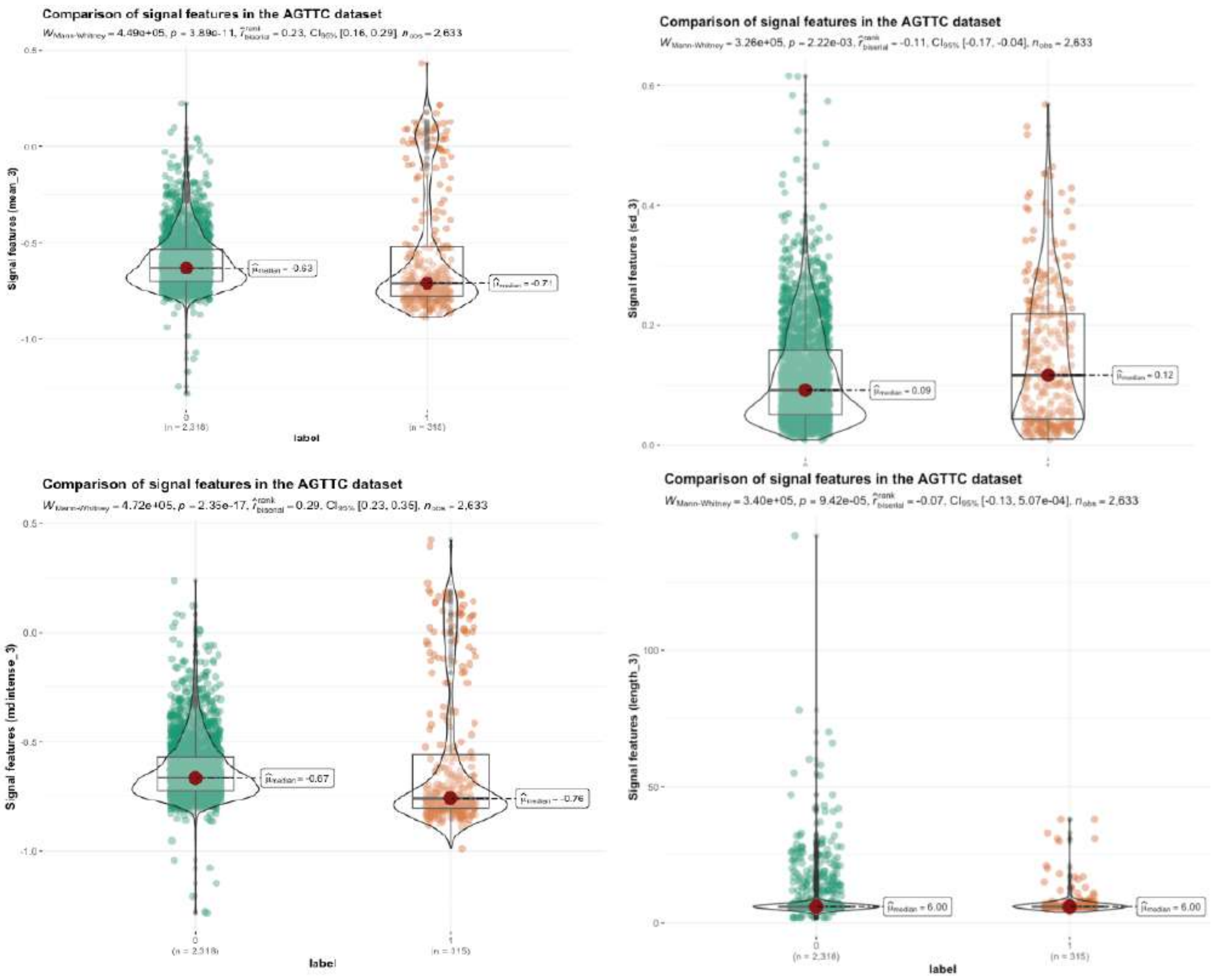

Figure S2

1.(d) AGTTC dataset: Signal features in the fourth position

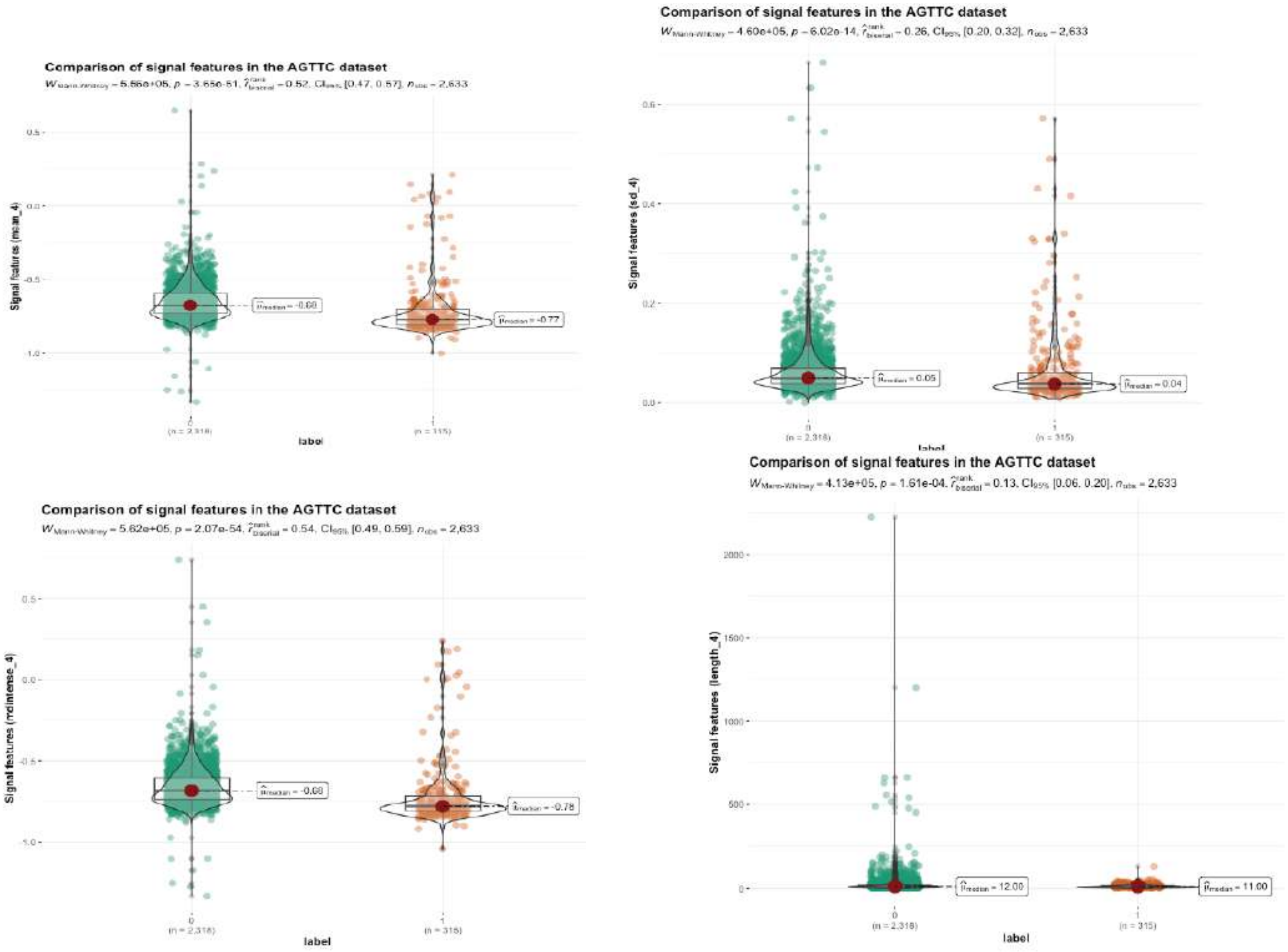

Figure S2-1.(e) AGTTC dataset: Signal features in the fifth position.

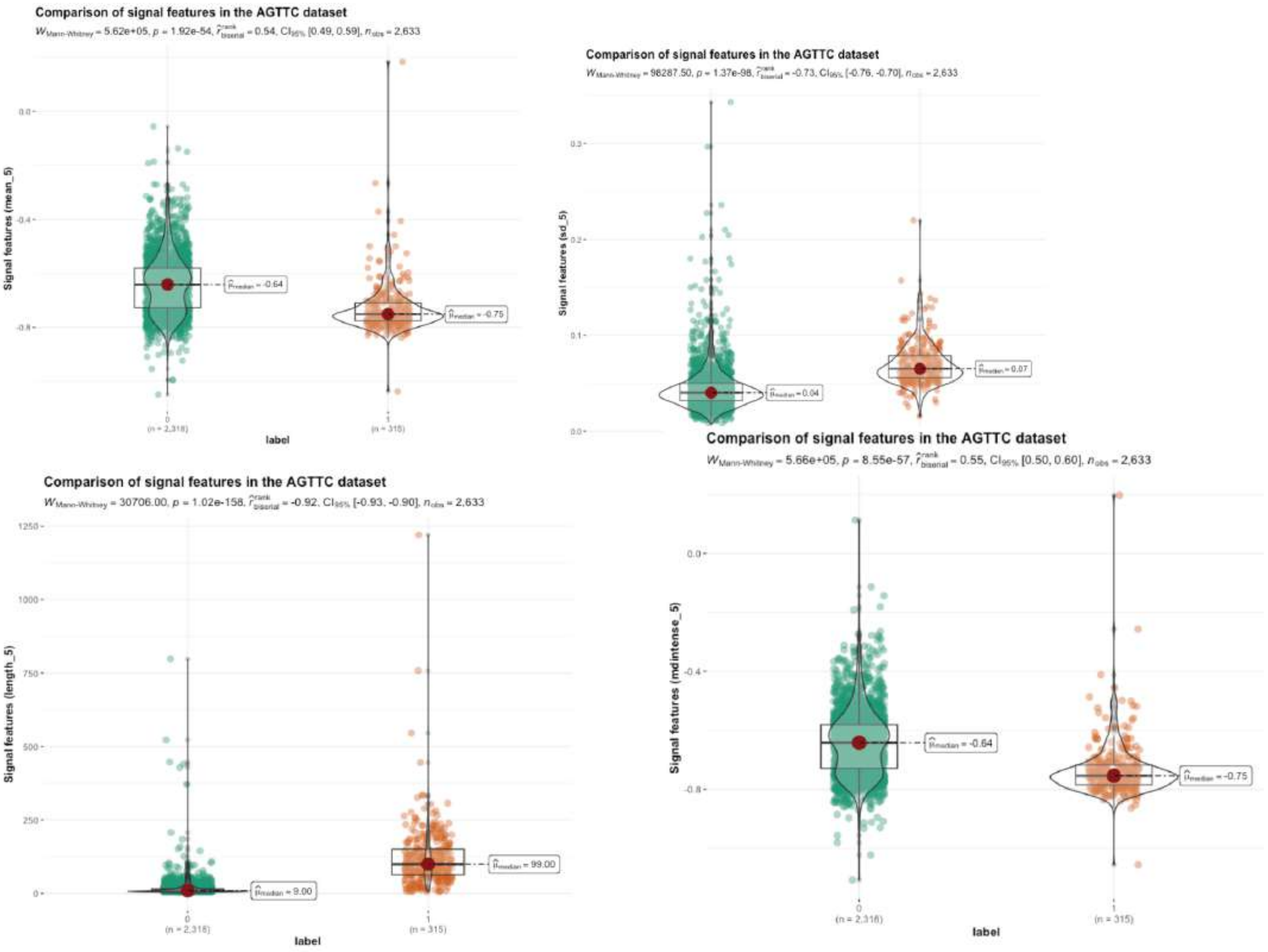

Figure S2 - 2. TGTGC result

2. (a) TGTGC dataset: Signal features in the first position.

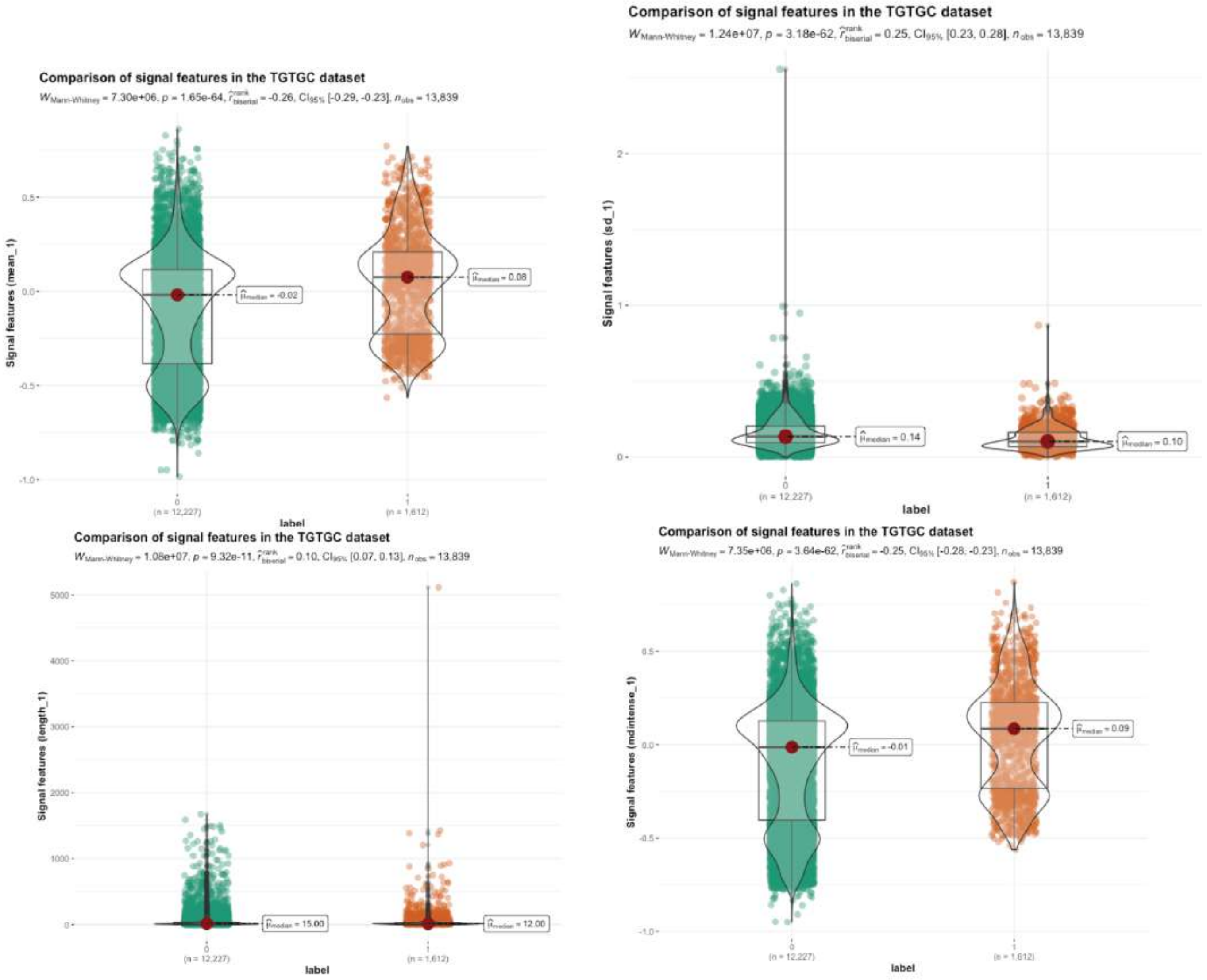

Figure S2 - 2.(b) TGTGC dataset: Signal features in the second position.

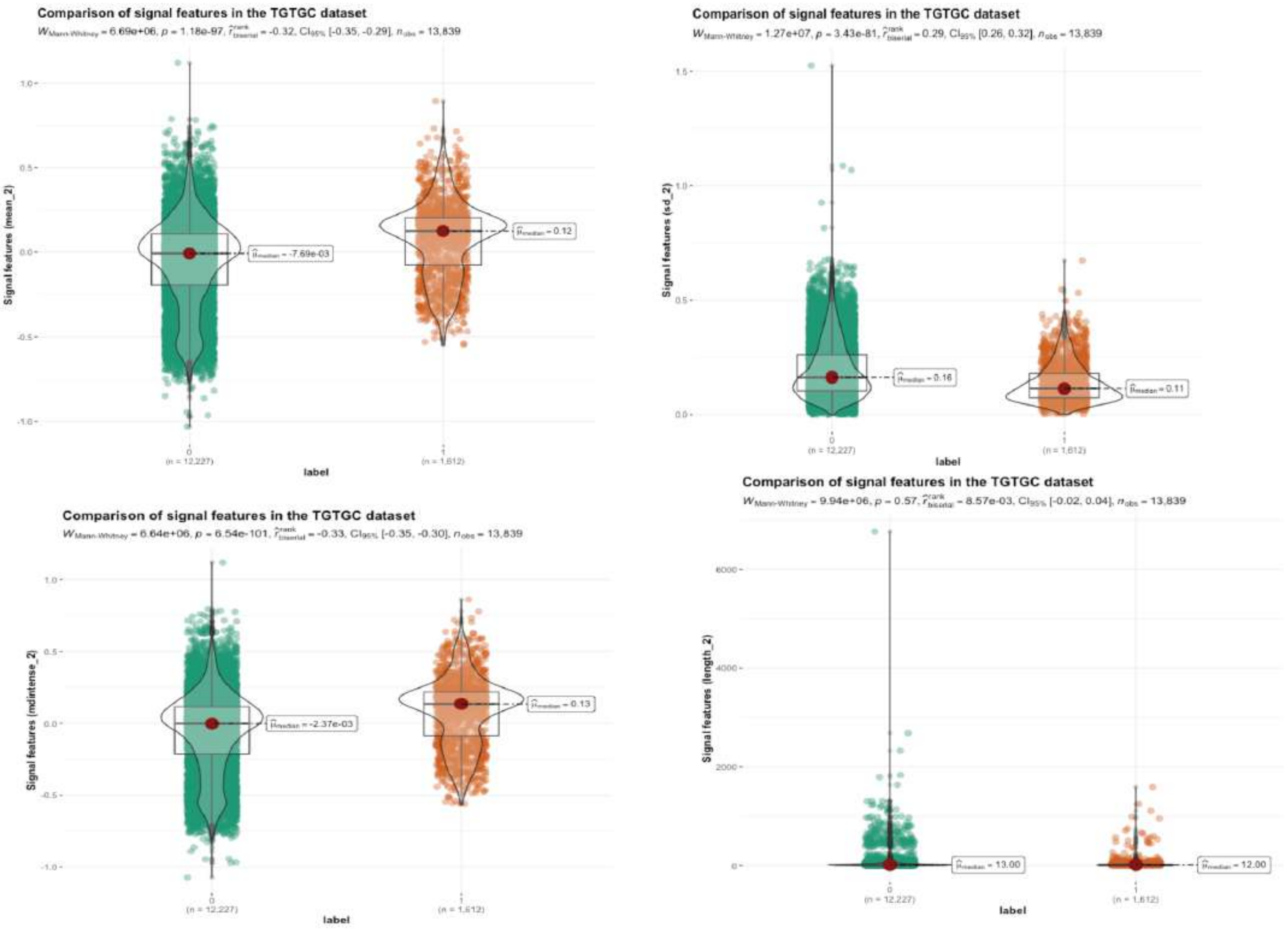

Figure S2 2. (c) TGTGC dataset: Signal features in the third position.

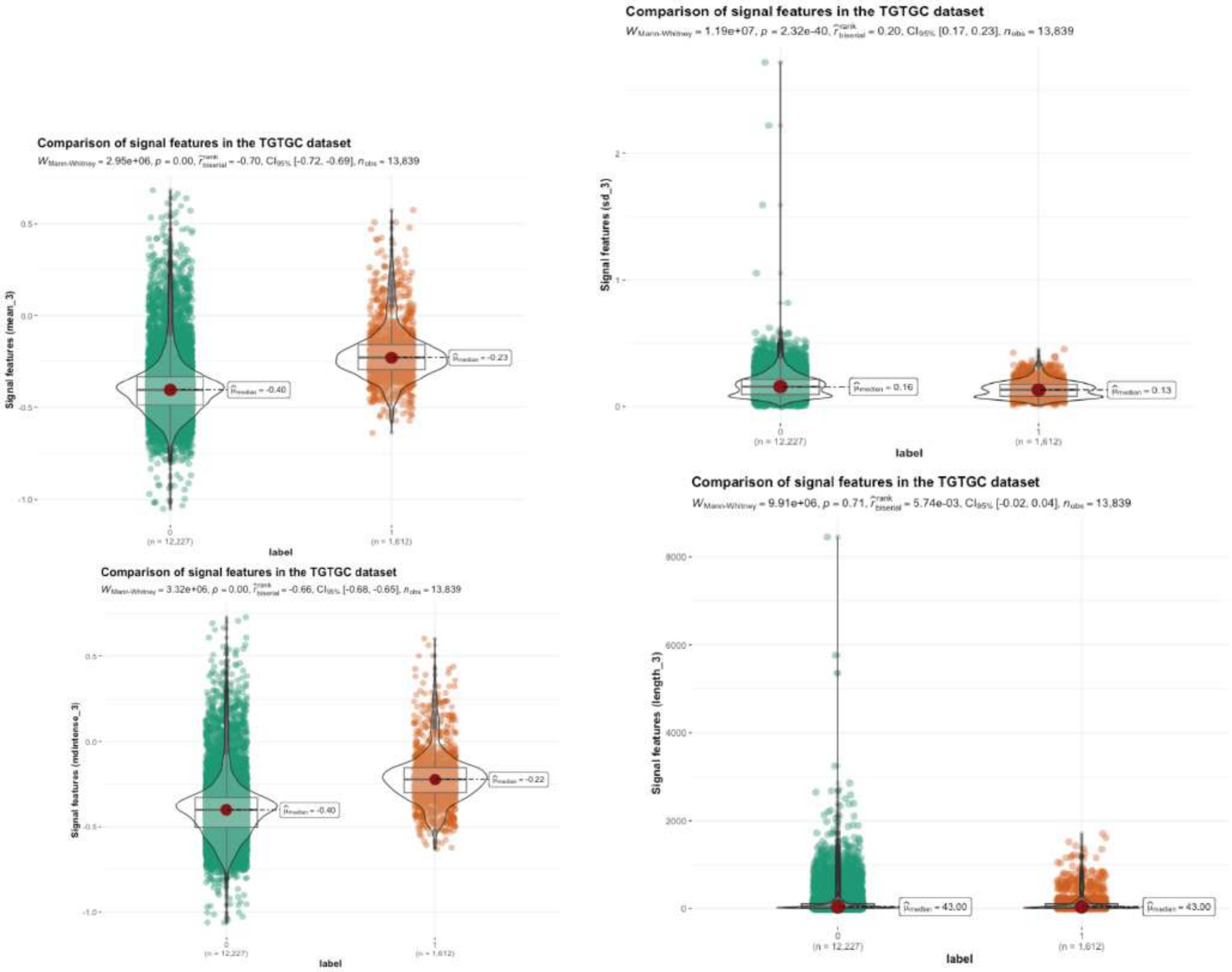

Figure S2 -2.(d) TGTGC dataset: Signal features in the fourth position.

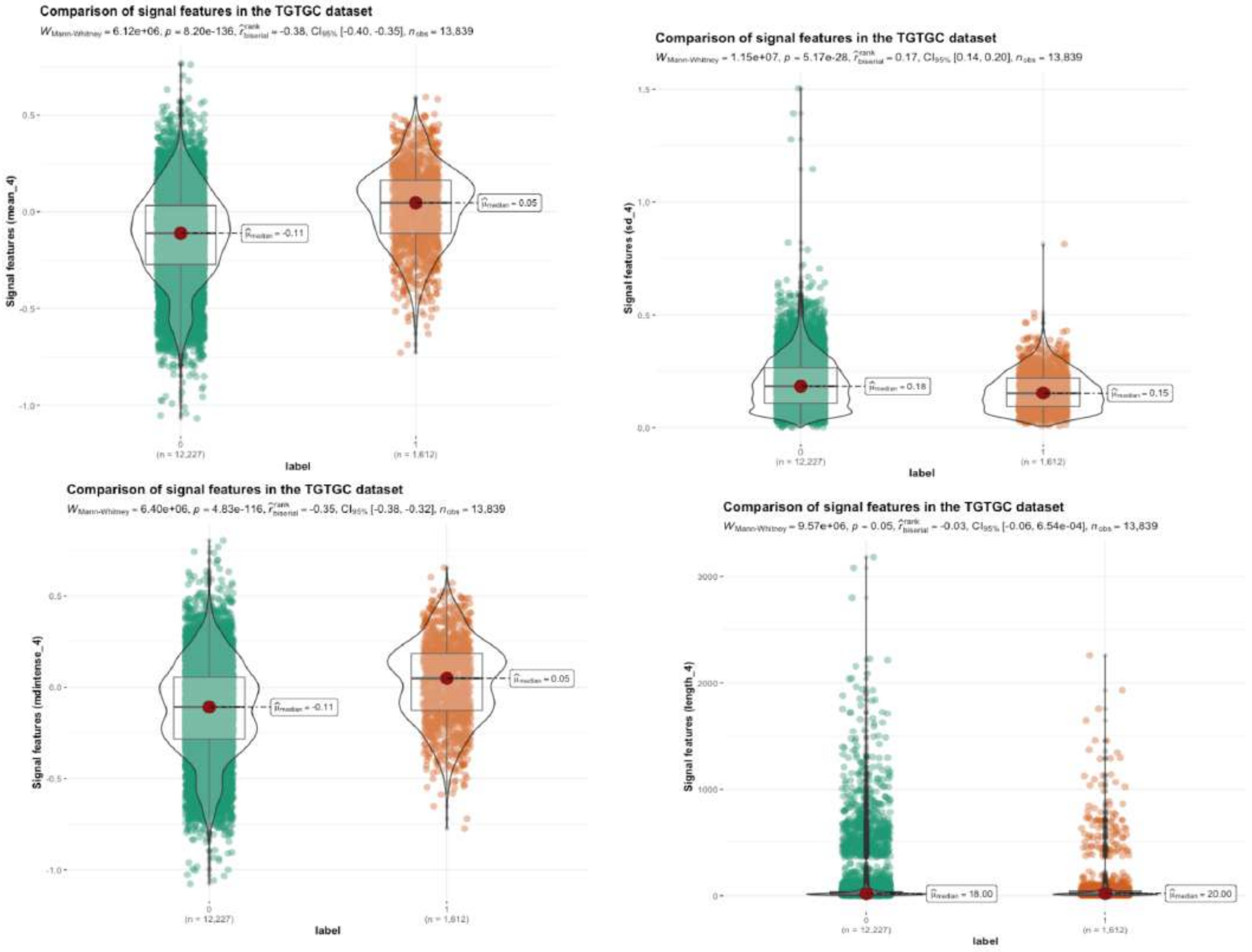

Figure S2 - 2.(e) TGTGC dataset: Signal features in the fifth position.

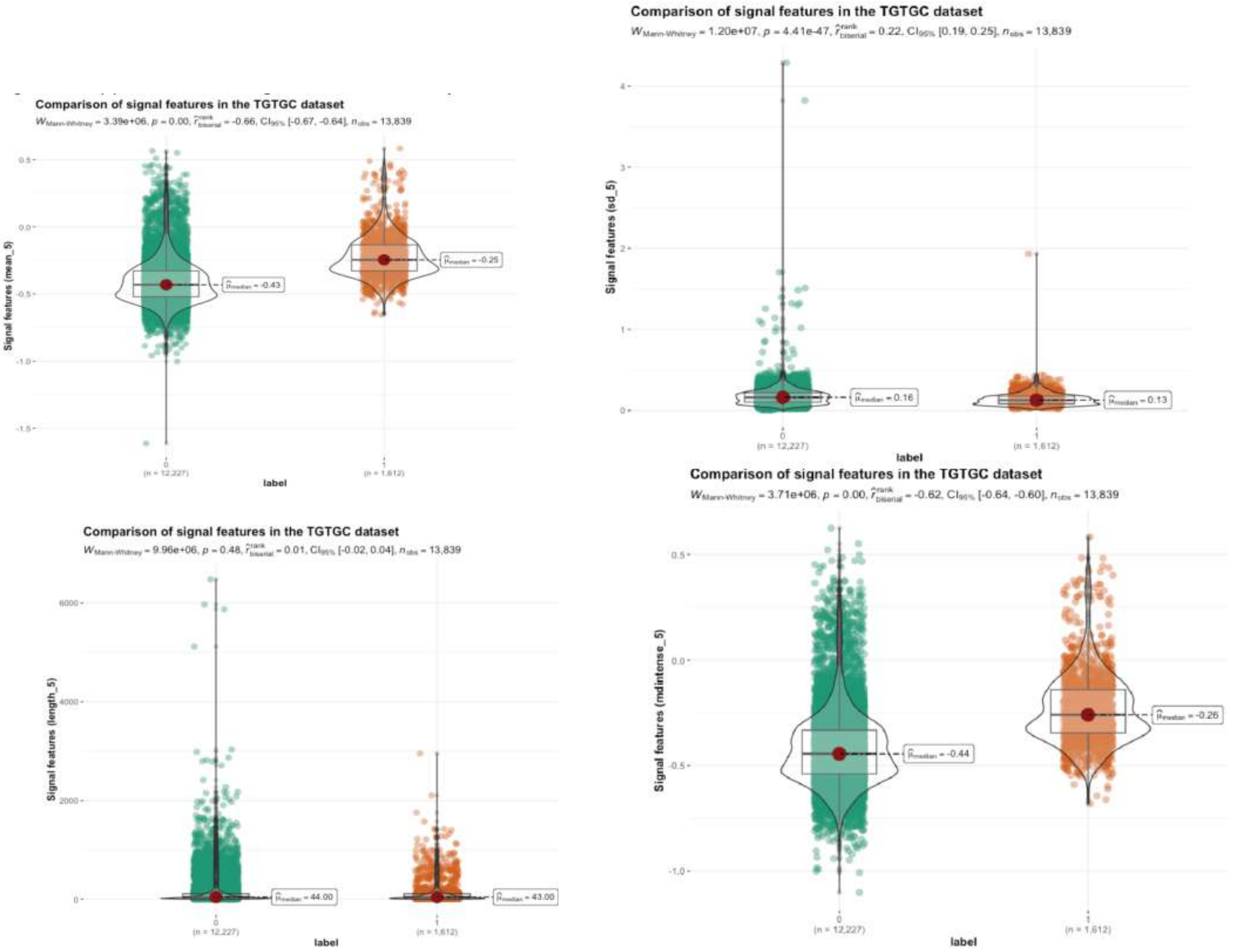

Figure S3. The feature contribution of the XGBoost model is visualized by the SHAP function.

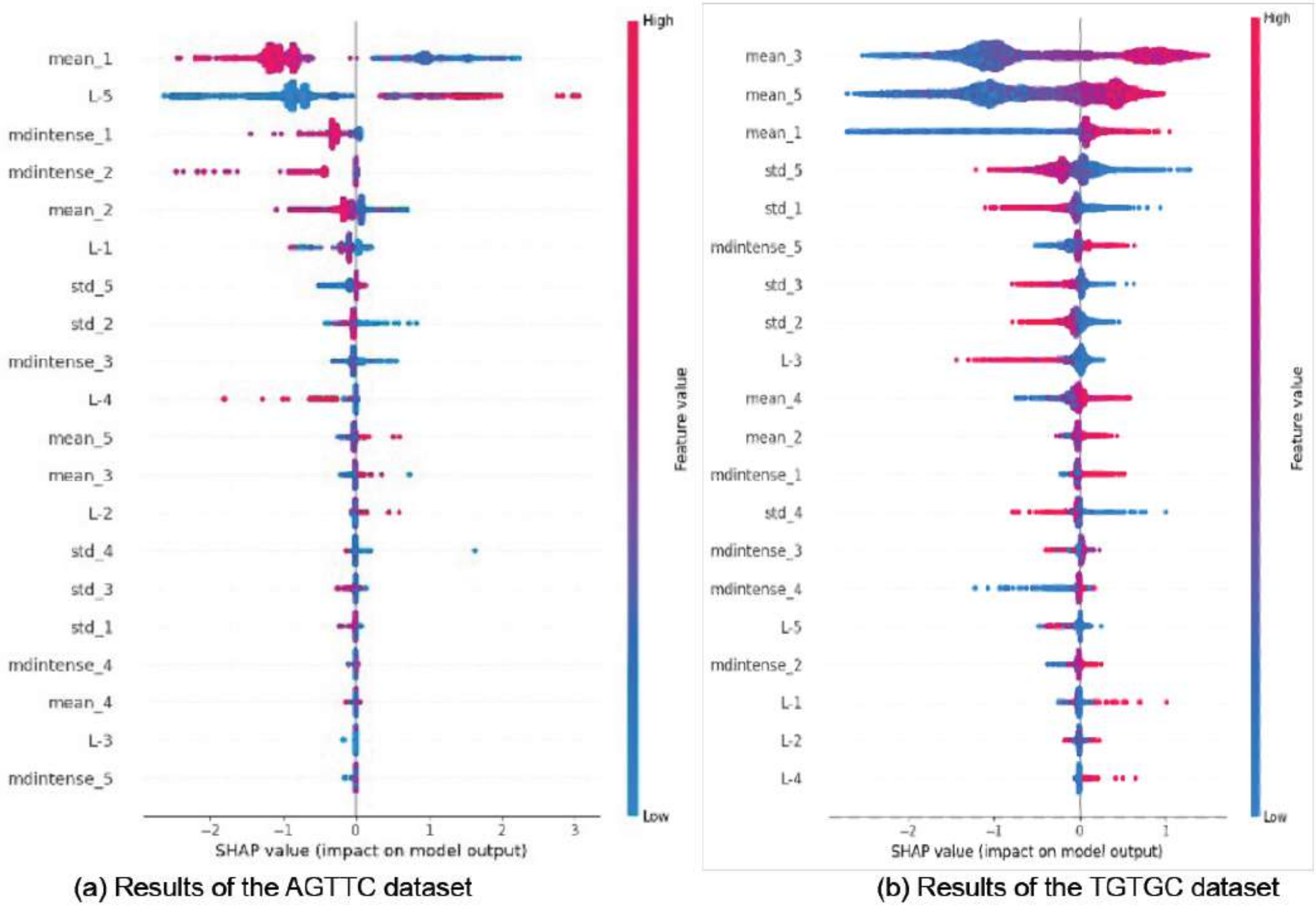

Figure S4. The complete Visualization of the read-level modification probability across sites of the given transcript based on three machine learning models on the AGTTC dataset.

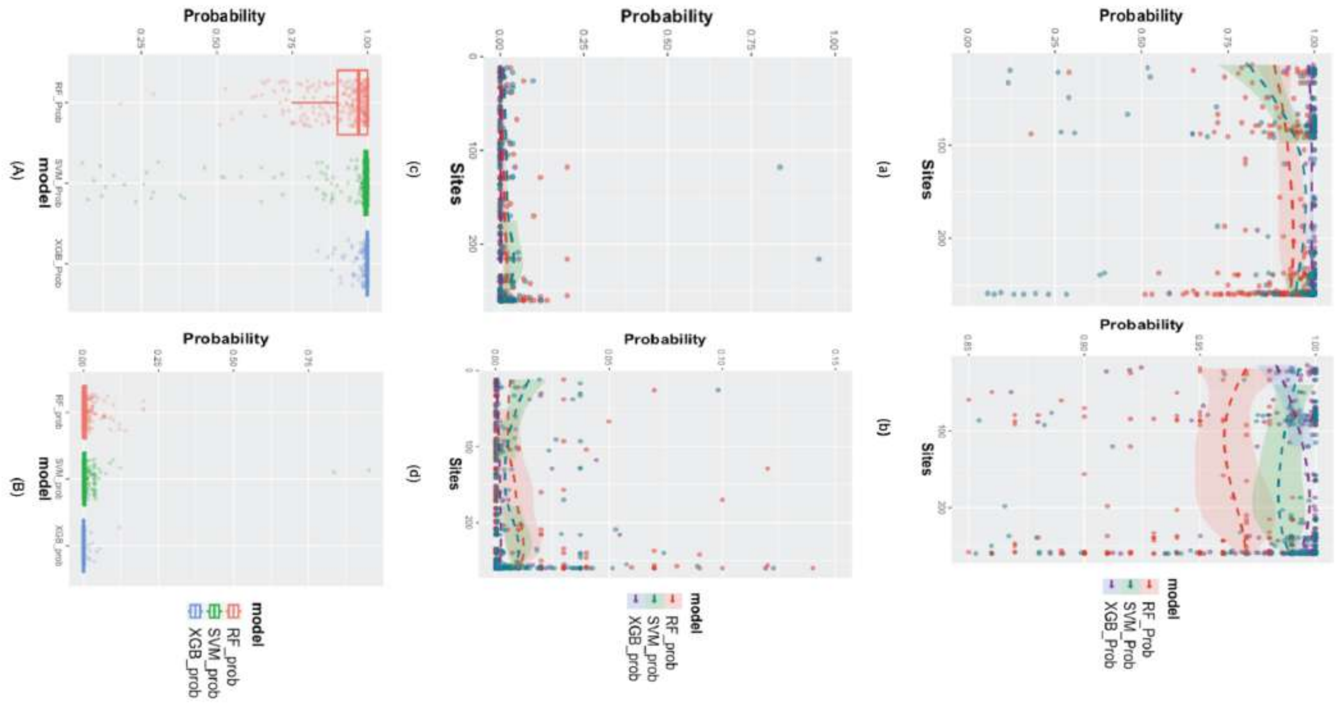

Figure S5. The complete Visualization of the read-level modification probability across sites of the given transcript based on three machine learning models on the TGTGC dataset.

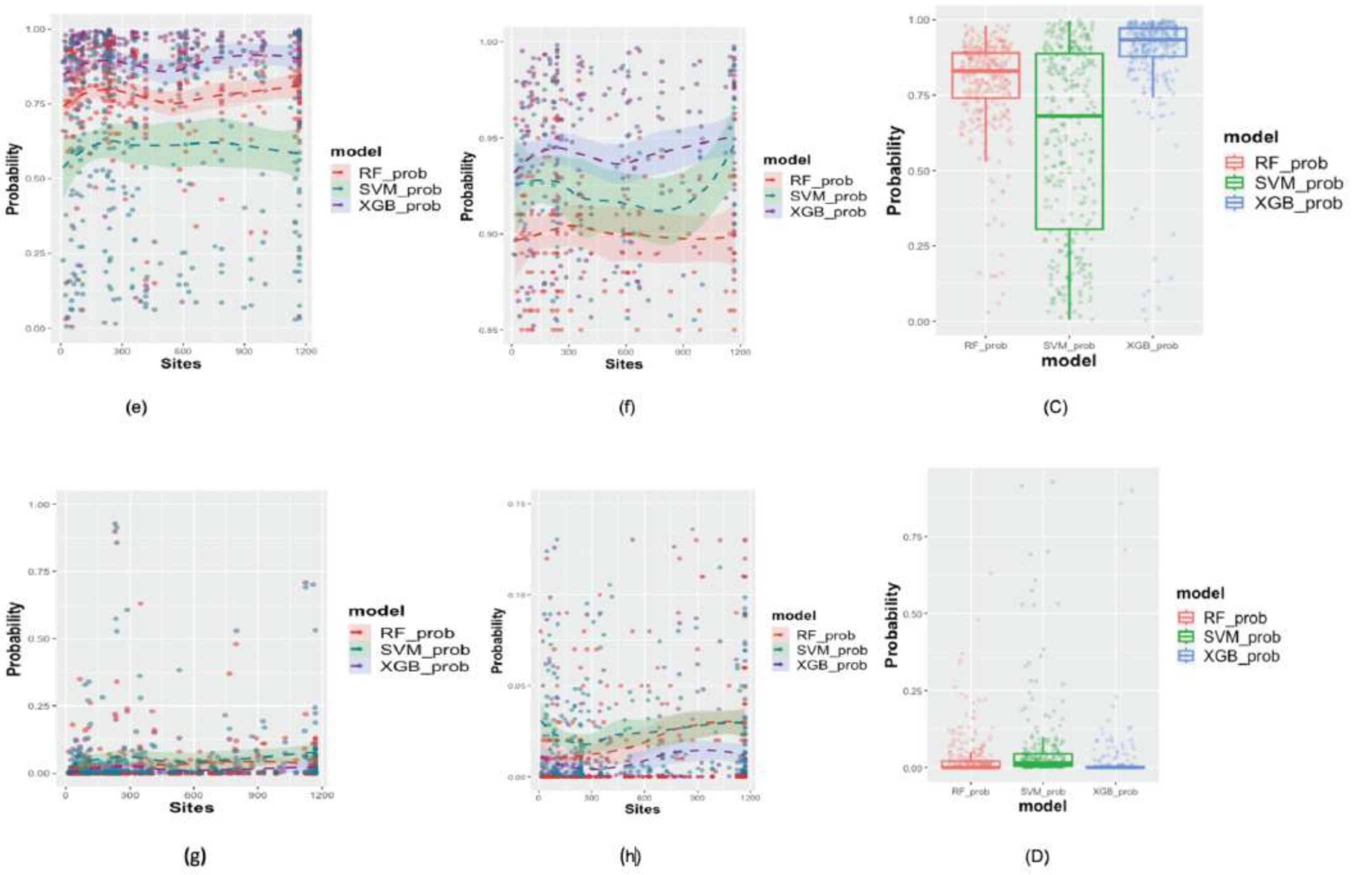

Supplement: Supplementary file 1 [file CG-25-212_SD1.pdf]
